# Prediction of COVID‐19 severity using machine learning

**DOI:** 10.1002/ctm2.70042

**Published:** 2024-10-06

**Authors:** Kanita Karaduzovic‐Hadziabdic, Muhamed Adilovic, Lu Zhang, Andrew I Lumley, Pranay Shah, Muhammad Shoaib, Venkata Satagopam, Prashant Kumar Srivastava, Costanza Emanueli, Simona Greco, Alisia Madè, Teresa Padro, Pedro Domingo, Mitja Lustrek, Markus Scholz, Maciej Rosolowski, Marko Jordan, Bettina Benczik, Bence Ágg, Péter Ferdinandy, Andrew H Baker, Guy Fagherazzi, Markus Ollert, Joanna Michel, Gabriel Sanchez, Hüseyin Firat, Timo Brandenburger, Fabio Martelli, Lina Badimon, Yvan Devaux, COVIRNA consortium (www.covirna.eu)

**Affiliations:** ^1^ Faculty of Engineering and Natural Sciences International University of Sarajevo Sarajevo Bosnia and Herzegovina; ^2^ Bioinformatics Platform Luxembourg Institute of Health Strassen Luxembourg; ^3^ Cardiovascular Research Unit Department of Precision Health Luxembourg Institute of Health Strassen Luxembourg; ^4^ Department of Epidemiology and Biostatistics School of Public Health Imperial College London London UK; ^5^ Luxembourg Center for Systems Biomedicine University of Luxembourg Belval Luxembourg; ^6^ National Heart and Lung Institute Imperial College London London UK; ^7^ Molecular Cardiology Laboratory IRCCS Policlinico San Donato Milan Italy; ^8^ Cardiovascular Program‐ICCC Institut d'Investigació Biomèdica Sant Pau (IIB SANT PAU); CIBERCV; Autonomous University of Barcelona Barcelona Spain; ^9^ Department of Intelligent Systems Jozef Stefan Institute Ljubljana Slovenia; ^10^ Group Genetical Statistics and Biomathematical Modelling Institute for Medical Informatics Statistics and Epidemiology University of Leipzig Leipzig Germany; ^11^ Cardiometabolic and HUN‐REN–SU System Pharmacology Research Group Department of Pharmacology and Pharmacotherapy Semmelweis University Budapest Hungary; ^12^ Center for Pharmacology and Drug Research & Development Semmelweis University Budapest Hungary; ^13^ Pharmahungary Group Szeged Hungary; ^14^ Centre for Cardiovascular Science The Queen's Medical Research Institute University of Edinburgh Edinburgh Scotland UK; ^15^ Department of Pathology CARIM Maastricht University Maastricht The Netherlands; ^16^ Deep Digital Phenotyping Research Unit Department of Precision Health Luxembourg Institute of Health Strassen Luxembourg; ^17^ Department of Infection and Immunity Luxembourg Institute of Health Esch‐Sur‐Alzette Luxembourg; ^18^ Firalis SA Huningue France; ^19^ Department of Anesthesiology University Hospital Düsseldorf Heinrich‐Heine University Duesseldorf Moorenstr Germany

Dear Editor,

Prediction of COVID‐19 severity is a critical task in the decision‐making process during the initial stages of the disease, enabling personalised surveillance and care of COVID‐19 patients. To develop a machine learning (ML) model for the prediction of COVID‐19 severity, a consortium of 15 institutions from 12 European countries analysed expression data of 2906 blood long noncoding RNAs (lncRNAs) and clinical data collected from four independent cohorts, totalling 564 patients with COVID‐19. This predictive model based on age and five lncRNAs predicted disease severity with an area under the receiver operating characteristic curve (AUC) of .875 [.868–.881] and an accuracy of .783 [.775–.791].

The sudden onset of the COVID‐19 pandemic caught the world unprepared, leading to more than 774 million confirmed cases and over 7 million reported deaths worldwide (over a period from January 2020 to March 2024), according to the World Health Organization (WHO).^1^ Other than having an impact on the respiratory system, severe acute respiratory syndrome coronavirus 2 (SARS‐CoV‐2) can also infect nonpulmonary cells such as cardiac and brain cells leading to cardiovascular or neurological symptoms.^2^ With the recent advances in high throughput sequencing, a large number of RNA signatures have emerged as promising biomarkers involved in the progression of various diseases, including cardiovascular diseases.^3^ As a response to the COVID‐19 pandemic, partners of the EU‐CardioRNA COST Action network^4^, ^5^, ^6^ joined forces in the H2020‐funded COVIRNA project to develop an RNA‐based diagnostic test using artificial intelligence (AI) that can help predict clinical outcomes after COVID‐19.^7^ We chose to implement a targeted sequencing approach using the FIMICS panel of 2906 cardiac‐enriched or heart failure‐associated lncRNAs previously characterised by our consortium.^8^ In the present study, we aimed to apply the FIMICS panel to identify lncRNAs that will predict disease severity of COVID‐19 patients. We used an approach based on ML to conduct the predictive analysis, as ML algorithms are suitably capable of analysing the complex relationships between biomedical data.^9^

The overall workflow of the study is illustrated in Figure 1A. Briefly, four European cohorts were included in the study consisting of a total of 564 patients with COVID‐19: the PrediCOVID cohort from Luxembourg (*n* = 162; recruitment period May 2020 to present), the COVID19_OMICS‐COVIRNA cohort from Italy (*n* = 100; recruitment period March 2020 to January 2021), the TOCOVID cohort from Spain (*n* = 233; recruitment period April 2020 to June 2021), and the MiRCOVID cohort from Germany (*n* = 69; recruitment period April 2020 to November 2021). Patient characteristics are presented in Table 1. Plasma samples collected from patients at baseline were stored at −80°C in a central NF S96‐900‐certified Biobank at Firalis SA. Samples were then processed using the following workflow: RNA extraction, quality check, library preparation, and analysis by targeted sequencing using the FIMICS panel. Overall, 463 datasets representing each unique patient from four independent cohorts were available for the present analysis (Figure 1B).

**FIGURE 1:**
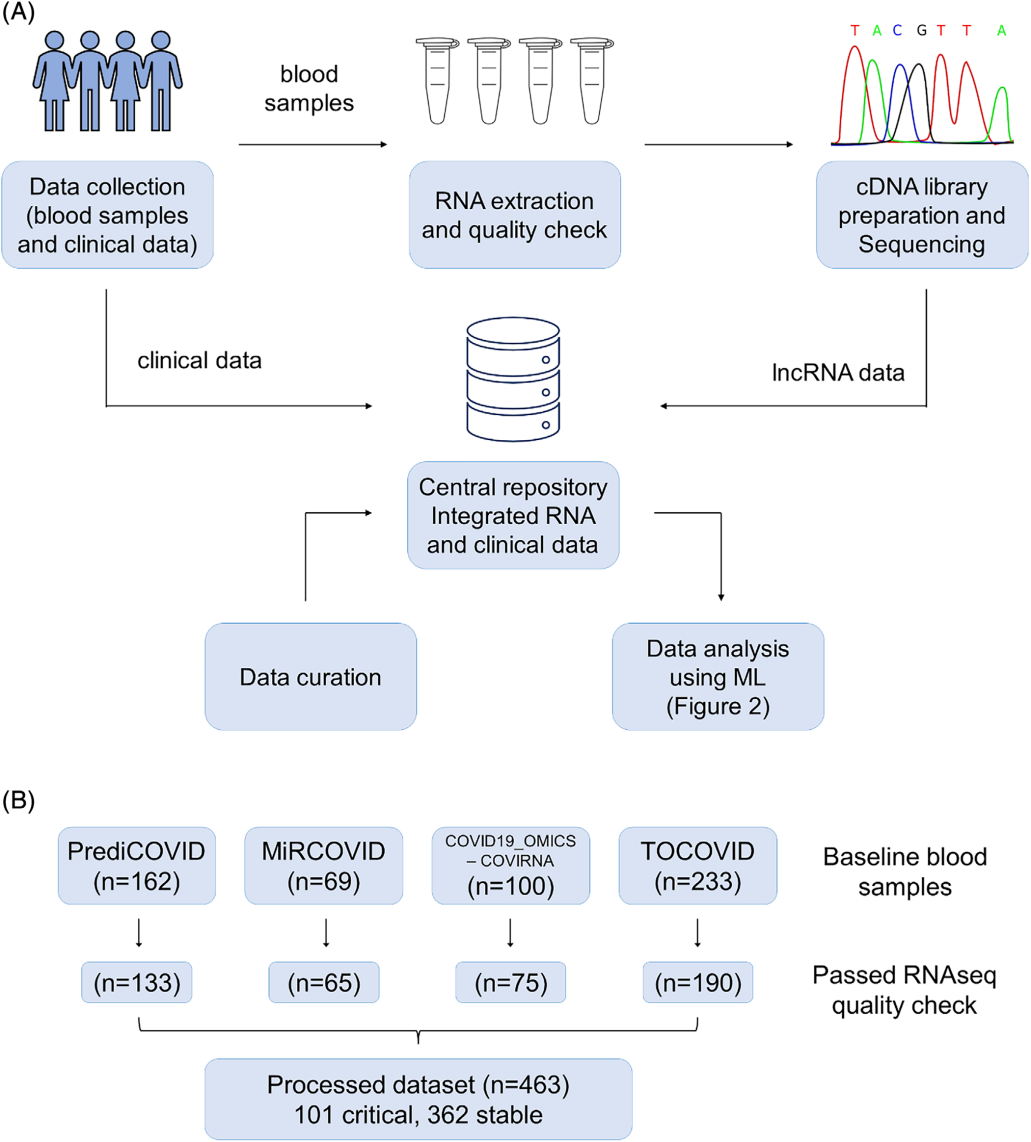
Study workflow and data available for the analysis (A) Study workflow. Blood samples stored at −80°C in a central NF S96‐900 certified Biobank at Firalis SA were collected from 564 patients with COVID‐19. Following this, RNA extraction, quality check, library preparation, and analysis by targeted sequencing using the FIMICS panel were performed. RNA seq data was then merged with patients’ clinical data and stored in a central database. Data was curated and made available for analysis using ML. (B) Baseline datasets available for analysis from four European cohorts: PrediCOVID from Luxembourg (*n* = 162), MiRCOVID from Germany (*n* = 69), COVID19_OMICS‐COVIRNA from Italy (*n* = 100), and TOCOVID from Spain (*n* = 233). Patient numbers indicated for each cohort after data curation and preprocessing: PrediCOVID from Luxembourg (*n* = 133), MiRCOVID from Germany (*n* = 65), COVID19_OMICS‐COVIRNA from Italy (*n* = 75), and TOCOVID from Spain (*n* = 195). A total of 463 datasets were available for the analysis.

**TABLE 1:** Characteristics of patients in the study cohort.

	All (*n* = 463)	Critical (*n* = 101)	Stable (*n* = 362)	*p* (critical vs. stable)
Age (mean ± SD)	53 ± 15.9	64.3 ± 14.3	49.8 ± 14.9	3.86E‐17
BMI (median [min, max])	27.5 [14.4, 57.8]	27.8 [14.4, 53.3]	27.3 [18.4, 57.8]	6.34E‐02
Sex_male (*n* (%))	275 (59.40)	72 (71.29)	203 (56.08)	6.00E‐03
Smoker_current (*n* (%))	47 (10.15)	12 (11.88)	35 (9.67)	5.76E‐01
Smoker_ex (*n* (%))	59 (12.74)	9 (8.91)	50 (13.81)	2.38E‐01

BMI, body mass index; SD, standard deviation.

The 463 datasets were then used in a ML workflow to identify the most important predictors (lncRNAs and clinical variables) and to build a model predicting disease severity of COVID‐19 patients in balanced (Figure 2A) and imbalanced (Figure 2B) datasets. Briefly, the available data was split into training and validation sets (80/20 split), then feature selection was performed on the training set—for features to be selected they had to appear in 90 out of the 100 iterations. The selected features were included in a model which was then evaluated using the validation set before the final model with the highest predictive capacity (highest AUC) was chosen. Using the described method, we identified six features as best predictors of COVID‐19 severity which were selected in more than 90 out of 100 iterations (Figure 3A). Cross‐validation of the selected features was also performed using 2 biostatistical methods (GLMnet and Stability selection; Figure 3B). The six features identified were age and five lncRNAs: SEQ0548 (LINC01088‐201), SEQ0817 (FGD5‐AS1), SEQ1056 (LINC01088‐209), SEQ3051 (an unannotated lncRNA, henceforth referred to as lncCOVIRNA1) and SEQ1321 (AKAP13‐SI). Box/violin plots of the selected predictors (Figure 4A–F) show significant (*p* < .001) differences between patients in the critical and stable groups.

**FIGURE 2:**
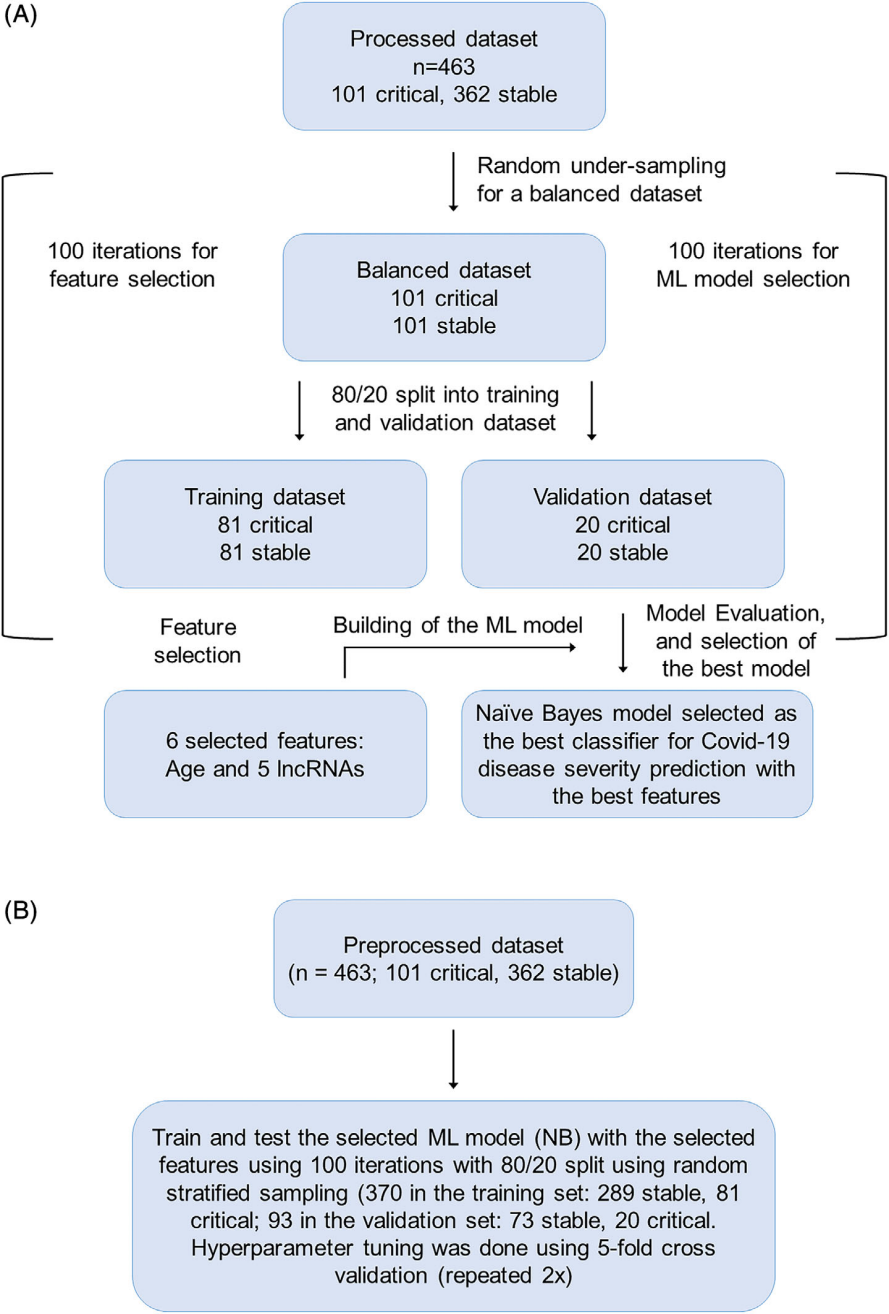
Machine learning workflow. Machine learning workflow using (A) balanced dataset and (B) imbalanced dataset.

**FIGURE 3:**
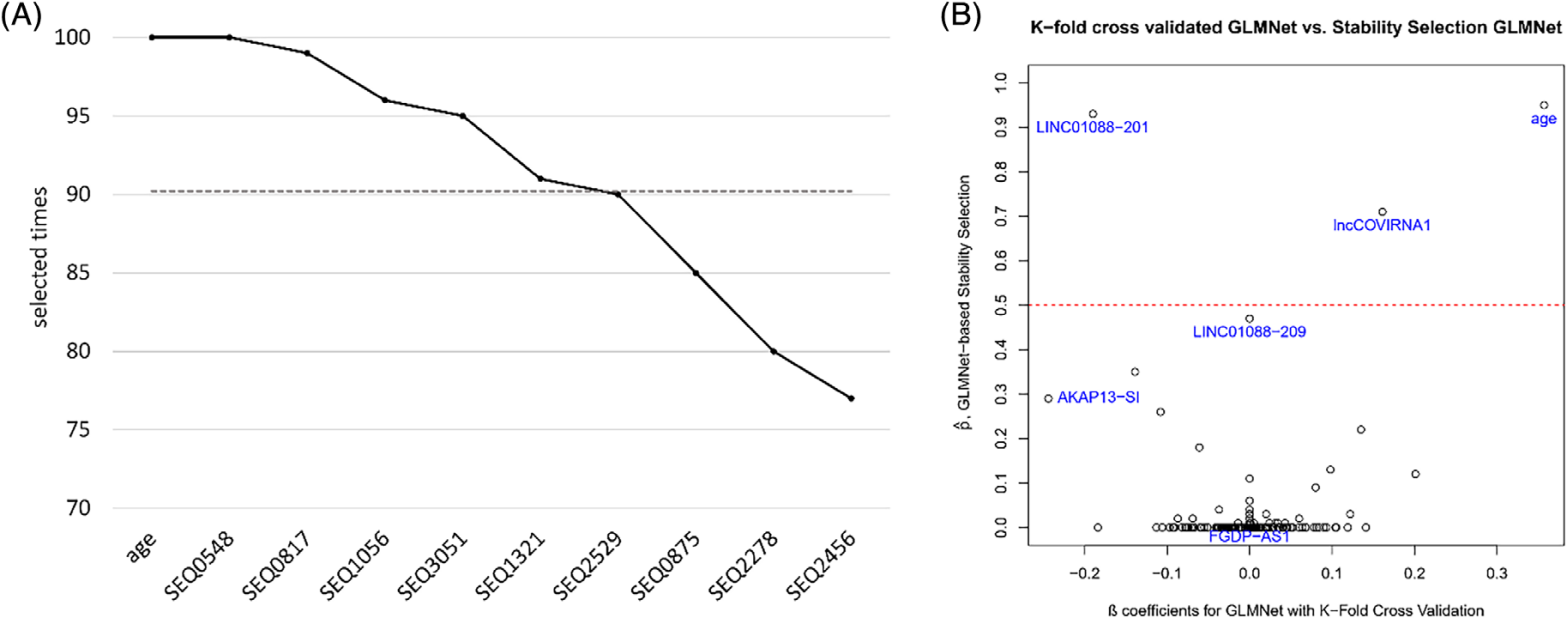
Feature selection. (A) Six features were selected as best predictors of COVID‐19 severity in more than 90 out of 100 iterations: age, SEQ0548 (LINC01088‐201), SEQ0817 (FGD5‐AS1), SEQ1056 (LINC01088‐209), SEQ3051 (lncCOVIRNA1), and SEQ1321 (AKAP13‐SI). The line plot shows the top 10 selected features. *X*‐axis: feature names: SEQXXXX is the code of the probe of the FIMICS panel. SEQ0548 and SEQ1056 probes recognise two different isoforms of the same gene LINC01088 (the former LINC01088‐201, and latter LINC01088‐209), SEQ0817 recognises FGD5‐AS1, SEQ3051 recognises an unannotated lncRNA (i.e. lncCOVIRNA1), and SEQ1321 recognises AKAP13‐SI. Y‐axis: the number of times a feature appeared in the 100 iterations of the feature selection process. (B) GLMNet and SS methods used to cross‐validate the selected features. The probability of selection of predictors plotted against the values of the regression coefficients (*ß*) for the leave‐one‐out cross‐validated GLMNet model. Each point represents a unique predictor. In the plot, the *X*‐axis represents the values of the regression coefficients of the predictors, where nonzero values indicate selection by the GLMNet model. The *Y*‐axis represents the frequentist probability of predictor selection when running a SS model. The probabilities of the features selected by the Boruta method are as follows: age (.95), LINC01088‐201 (.93), lncCOVIRNA1 (.71), LINC01088‐209 (.47), AKAP13‐SI (.29) and FGD5‐AS1 (.01).

**FIGURE 4:**
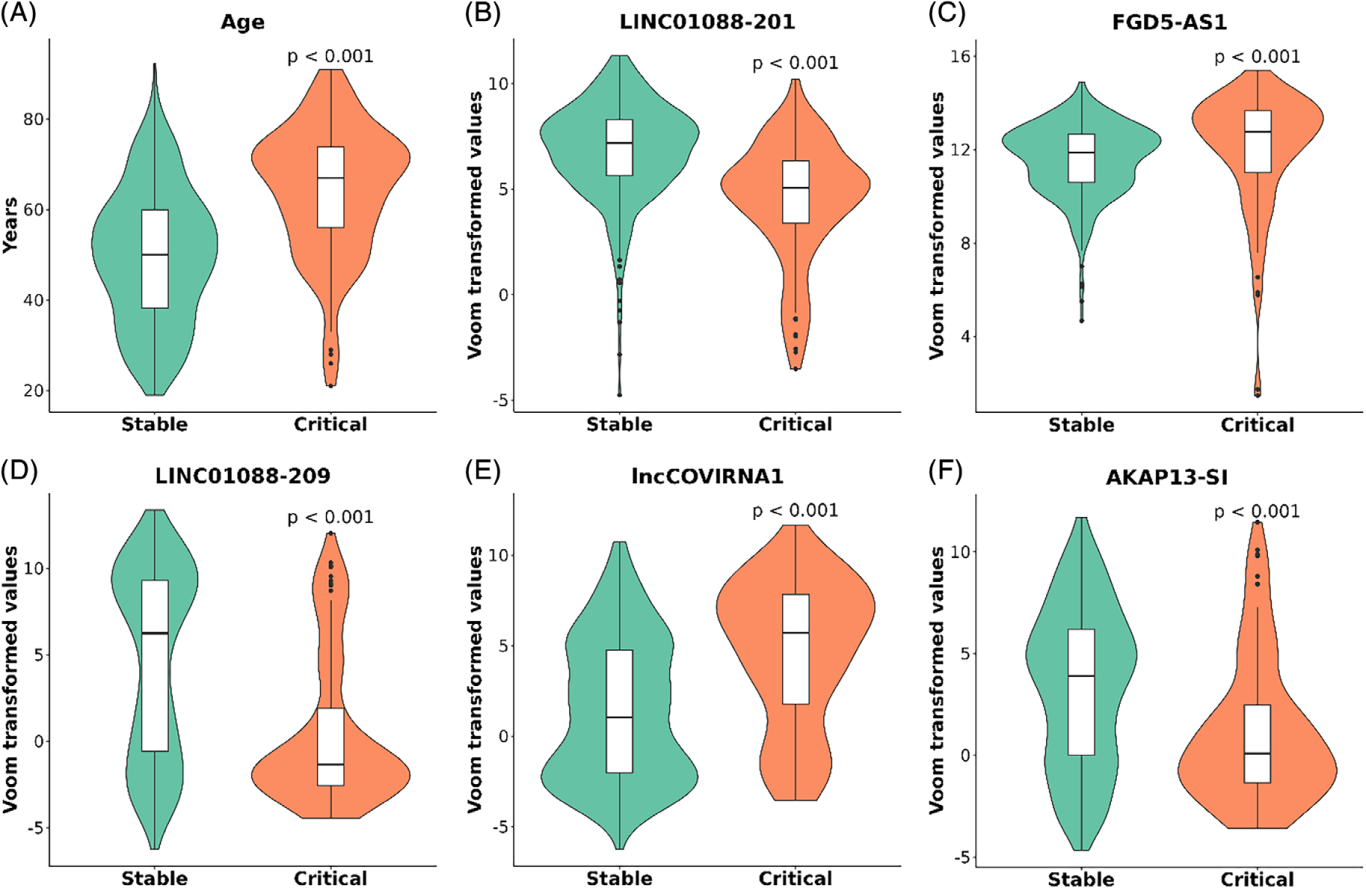
Comparison of selected features between stable and critical patients. Box/violin plots for (A) age, and expression of: (B) LINC01088‐201, (C) FGD5‐AS1, (D) LINC01088‐209, (E) lncCOVIRNA1, and (F) AKAP13‐SI showing regulations in the critical group of the merged cohort (*n* = 101) as compared to the group of stable patients (*n* = 362). *p* Value is from Student's *t* test. Boxes are drawn from Q1 (25th percentile) to Q3 (75th percentile) with a horizontal line inside it to denote the median. The length of the whiskers indicates 1.5 times of IQR (interquartile range Q3–Q1).

Table 2 presents results on the balanced dataset using the six selected features (age, LINC01088‐201, FGD5‐AS1, LINC01088‐209, lncCOVIRNA1 and AKAP13‐SI) across multiple ML models (Naïve Bayes, Logistic Regression, Extreme gradient boosting, Support Vector Machine, Multilayer Perceptron, K‐Nearest Neighbours). We also built and evaluated the performance of ML models using only age as a predictor (Table S1) and using only the five selected lncRNAs (Table S2). Overall, the best results were obtained using all six selected features (age and the five lncRNAs) in the Naïve Bayes model which allowed an AUC of .875 (95% CI .868–.881) and an accuracy of .783 (95% CI .775–.791, Table 2 and Figure S1).

**TABLE 2:** Performance of six ML models to predict COVID‐19 severity using selected features (age, LINC01088‐201, LINC01088‐209, lncCOVIRNA1, AKAP13‐SI and FGD5‐AS1) and the balanced dataset.

	AUC (95% CI)	Accuracy (95% CI)	Sensitivity (95% CI)	Specificity (95% CI)
**NB**	**.875 (.868–.881)**	**.783 (.775–.791)**	**.765 (.747–.784)**	**.788 (.778–.797)**
**LR**	.866 (.858–.873)	.769 (.762–.776)	.762 (.743–.782)	.771 (.761–.78)
**XGB**	.863 (.855–.87)	.775 (.766–.784)	.804 (.787–.822)	.767 (.755–.779)
**SVM**	.855 (.848–.863)	.751 (.742–.76)	.787 (.77–.805)	.740 (.728–.753)
**MLP**	.839 (.83–.848)	.762 (.754–.771)	.762 (.742–.782)	.762 (.752–.773)
**K‐NN**	.833 (.824–.841)	.751 (.742–.759)	.777 (.757–.797)	.744 (.733–.754)

*Note*: Bold indicates the best predictions

NB, Naïve Bayes; LR, logistic regression; XGBoost, extreme gradient boosting, SVM, support vector machine; MLP, multilayer perceptron; K‐NN, K‐nearest neighbours.

The developed ML model can be used as an integral part of the development of a molecular diagnostic assay utilising routinely available quantitative PCR methods to quantify blood levels of the five lncRNAs to be used as input to the ML model for COVID‐19 severity prediction. Together with another whole blood‐based ML algorithm,^10^ the use of the present ML model based on plasma samples could have significant clinical implications, for instance by selecting high‐risk patients for tailored treatment. An advantage of the present method is that it allows faster risk stratification of patients for decision making, which is especially useful during a pandemic, and is based on a widely used plasma sample. LncRNAs can be easily and quickly (2 h) measured in a noninvasive plasma sample. The increasing interest of the biomedical community on RNA molecules to treat or vaccinate patients could be followed by approval of circulating RNAs as disease biomarkers for personalised medicine, coupled with artificial intelligence methods.^7^ Moreover, identification of novel disease biomarkers could enhance our knowledge of the mechanisms leading to adverse outcomes or death, which could pave the way to the development of new therapies or repurposing of existing ones.

Taken together, these findings could have significant clinical value to predict disease severity and help to improve the management and outcomes of COVID‐19 patients.

## AUTHOR CONTRIBUTIONS

All authors are members of the COVIRNA consortium who conducted the present study. Kanita Karaduzovic‐Hadziabdic, Muhamed Adilovic, Fabio Martelli, Yvan Devaux and Lu Zhang designed the research study. Yvan Devaux acquired funding. Kanita Karaduzovic‐Hadziabdic, Muhamed Adilovic and Lu Zhang conducted the experiments. Pranay Shah conducted GLMNet and SS analysis. Muhammad Shoaib curated the data. Lu Zhang and Muhamed Adilovic preprocessed the data. Kanita Karaduzovic‐Hadziabdic, Muhamed Adilovic, Yvan Devaux, Lu Zhang, Andrew I Lumley, Pranay Shah, Muhammad Shoaib, Prashant Kumar Srivastava, Mitja Lustrek, Maciej Rosolowski, Marko Jordan and Bettina Benczik analysed data. Firalis staff (Joanna Michel, Gabriel Sanchez, Hüseyin Firat) were responsible for sample storage and RNA extraction, library preparation, targeted RNA sequencing and raw data analysis. Kanita Karaduzovic‐Hadziabdic wrote the draft and the final manuscript. Yvan Devaux supervised the writing of the manuscript and critically revised it for important intellectual content. Muhamed Adilovic, Yvan Devaux, Andrew I Lumley, Pranay Shah, Hüseyin Firat and Joanna Michel revised the manuscript and provided comments and wrote parts of the final manuscript. Muhamed Adilovic and Lu Zhang prepared the figures and tables. Yvan Devaux, Fabio Martelli, Alisia Madè, Simona Greco, Lina Badimon, Teresa Padro, Pedro Domingo, Timo Brandenburger, Guy Fagherazzi and Markus Ollert participated in acquiring patient samples and data. All the authors revised draft manuscript and approved the final version of the manuscript.

MA is co‐first author, who together with KK‐H conducted the majority of the experiments and other contributions as noted above. The order among cVo‐first authors was assigned based on contributions.

## CONFLICT OF INTEREST STATEMENT

YD holds patents and licensing agreements related to the use of RNAs for diagnostic and therapeutic purposes (WO2018229046, licensed to Firalis SA, protecting the use of lncRNAs in the FIMICS panel used for RNAseq in the present paper; other patents and licenses are not related to the present work). YD is Scientific Advisory Board member of Firalis SA.

PF is the founder and CEO of Pharmahungary Group, a group of R&D companies.

LB declares to have acted as a SAB member of Sanofi, Ionnis, MSD and NovoNordisk; to have received speaker fees from Sanofi, Bayer and AB‐Biotics SA and to have founded the spin‐off Ivastatin Therapeutics S.L. (all unrelated to this work).

TP declares to have received speaker fees from AB‐Biotics SA and to be a co‐founder of the Spin‐off Ivastatin Therapeutics SL (all unrelated to this work).

MS received funding from Pfizer Inc. and from Owkin for projects not related to this research.

HF is the founder and owner of Firalis SA, a company commercialising the FIMICS panel. He holds patents and licenses for the use of RNAs as biomarkers and therapeutic targets.

All other authors declare no competing interests.

## FUNDING INFORMATION

This work was supported by the EU Horizon 2020 project COVIRNA awarded to YD (grant agreement # 101016072).

The Predi‐COVID study was supported by the Luxembourg National Research Fund (FNR) (Predi‐COVID, grant number 14716273), the André Losch Foundation and by European Regional Development Fund (FEDER, convention 2018‐04‐026‐21).

YD is funded by the EU Horizon 2020 project COVIRNA (grant agreement # 101016072), the National Research Fund (grants # C14/BM/8225223, C17/BM/11613033 and COVID‐19/2020‐1/14719577/miRCOVID), the Ministry of Higher Education and Research, and the Heart Foundation‐Daniel Wagner of Luxembourg.

FM is supported by the Italian Ministry of Health (Ricerca Corrente 2024 1.07.128, RF‐2019‐12368521 and POS T4 CAL.HUB.RIA cod. T4‐AN‐09), EU COVIRNA agreement #101016072, Next Generation EU PNRR M6C2 Inv. 2.1 PNRR‐MAD‐2022‐12375790 and PNRR/2022/C9/MCID/I8 FibroThera.

Horizon 2020 Framework Programme 101016072, André Losch Fondation, Heart Foundation‐Daniel Wagner of Luxembourg, Ministero della Salute POS T4 CAL.HUB.RIA cod. T4‐AN‐09, RF‐2019‐12368521, Ricerca Corrente 2024 1.07.128, Fonds National de la Recherche Luxembourg C14/BM/8225223, C17/BM/11613033, COVID‐19/2020‐1/14719577/miRCOVID, Next Generation EU, European Regional Development Fund FEDER, convention 2018‐04‐026‐21, Ministère de l'Education Nationale, de l'Enseignement Superieur et de la Recherche. P.F. and B.Á. were funded by project no. RRF‐2.3.1‐21‐2022‐00003 that has been implemented with the support provided by the European Union. The 2020‐1.1.5‐GYORSÍTÓSÁV‐2021‐00011 project was funded by the Ministry for Innovation and Technology with support from the National Research Development and Innovation Fund under the 2020‐1.1.5‐GYORSÍTÓSÁV call programme. This study was funded by the grant 2020‐1.1.6‐JÖVŐ‐2021‐00013 ( “Befektetés a jÖvŐbe” NKFIH). This project has received funding from the HUN‐REN Hungarian Research Network.

The ML code is available as a Supplementary File and is accessible on the GitHub repository at the following link https://github.com/madilovic/COVIRNA_plasma using ID: 118e2ccd07df8b10b7fc52df95ae11b52bb8216a.

## ETHICS STATEMENT

This study was performed in full compliance with the Declaration of Helsinki. The study involved four cohorts comprising COVID‐19‐positive patients aged 18 years and older from Luxembourg (PrediCOVID study), Italy (COVID19_OMICS—COVIRNA study), Spain (TOCOVID study), and Germany (MiRCOVID study). The Luxembourg PrediCOVID study was approved by the National Research Ethics Committee of Luxembourg (study Number 202003/07) and was registered under ClinicalTrials.gov (NCT04380987). The COVID19_OMICS—COVIRNA study was approved by the Institutional Ethics Committee of the San Raffaele Hospital (protocol number 75/INT/2020, 20/04/2020 and subsequent modification dated 16/12/2020) and was registered with ID NCT04441502. The TOCOVID study was approved by the Research Ethics Committee of the Hospital Santa Creu i Sant Pau, Barcelona (Ref number 21/036) and was registered under ClinicalTrials.gov (NCT04332094). The MiRCOVID study was approved by the Research Ethics Committee of Duesseldorf University (internal study number 2020−912) and was registered under ClinicalTrials.gov (NCT04381351). Periods of patient enrolment and biological samples collection were May 2020 to present for PrediCOVID, March 2020 to January 2021 for COVID19_OMICS‐COVIRNA, April 2020 to June 2021 for TOCOVID, April 2020 to November 2021. Informed consent was signed by all patients enrolled in these studies. Legal agreements for material and data sharing have been signed between each cohort and COVIRNA project coordinator Luxembourg Institute of Health (LIH).

## Supporting information



Supporting information

Supporting information

## Data Availability

Due to legal and ethical issues related to General Data Protection Regulation guidelines, the data used in this study is available upon request to the COVIRNA consortium. Please email the corresponding author for more details and information about data access (yvan.devaux@lih.lu).
