# Supplementary material for: Prediction of COVID‐19 severity using machine learning
Source: Clin Transl Med. 2024 Oct 6;14(10):e70042. doi: 10.1002/ctm2.70042 (PMC11456675; doi:10.1002/ctm2.70042)
Supplement: Supplementary file 1 — Supporting information [file CTM2-14-e70042-s001.docx]

**
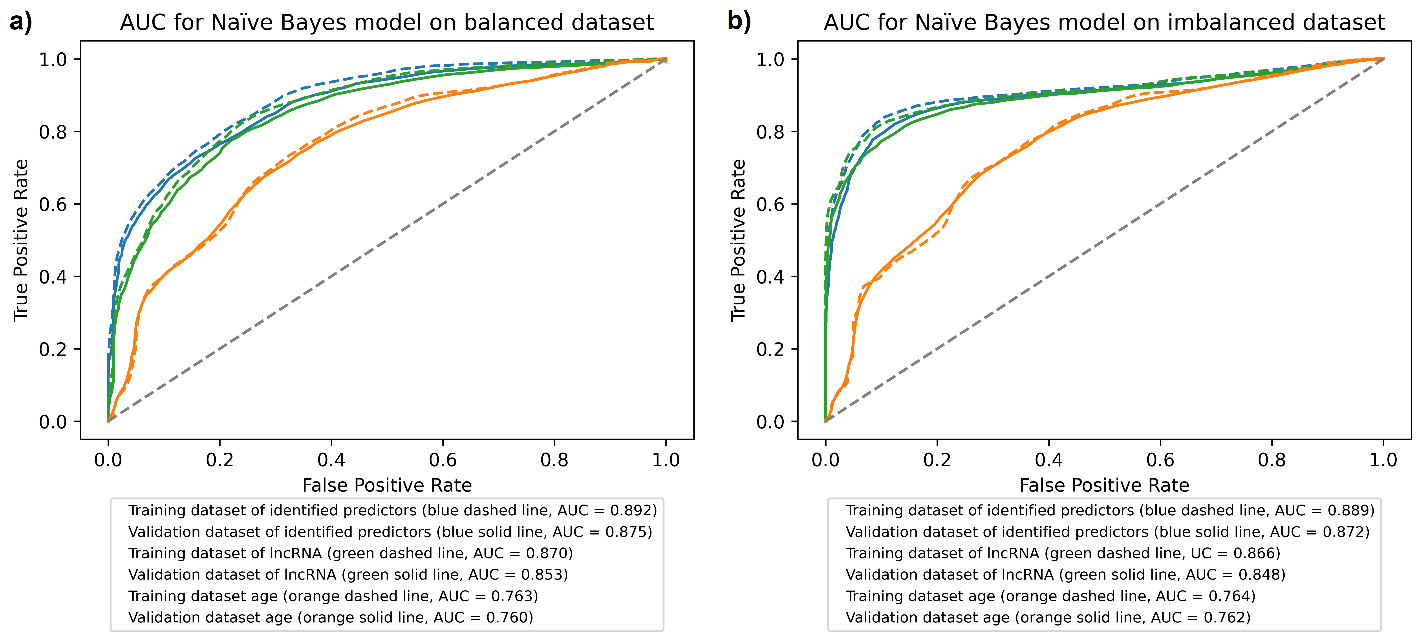
**

**Supplementary Figure 1.** **Receiver operating characteristic curves (ROC) of the NB model for training and validation datasets.** The ROC curves shown include all three data subsets: 1) using all selected features (age, LINC01088-201, FGD5-AS1, LINC01088-209, lncCOVIRNA1 and AKAP13-SI), 2) age only and 3) lncRNAs only, averaged across 100-iteration random sampling on **a)** balanced data training (n = 162) and validation (n = 40); **b)** imbalanced data training (n = 370) and validation (n = 93).
