# Supplementary material for: Prediction of COVID‐19 severity using machine learning
Source: Clin Transl Med. 2024 Oct 6;14(10):e70042. doi: 10.1002/ctm2.70042 (PMC11456675; doi:10.1002/ctm2.70042)
Supplement: Supplementary file 2 — Supporting information [file CTM2-14-e70042-s002.docx]

# Supplementary materials

Consortium

The following members of COVIRNA consortium who are not co-authors of this paper are listed below: Petr Nazarov, Luxembourg Institute of Health; Reinhard Schneider, University of Luxembourg; Nick Mills, University of Edinburgh; Ryan Wereski, University of Edinburgh; Eric Schordan, Firalis SA.

**Supplementary Table 1.** Outcome categories of study cohorts.

|  | Critical | Stable | Total |
| --- | --- | --- | --- |
| PrediCOVID | 0 | 133 | 133 |
| COVID19_OMICS-COVIRNA | 36 | 39 | 75 |
| TOCOVID | 0 | 190 | 190 |
| MiRCOVID | 65 | 0 | 65 |
| Merged study cohort | 101 | 362 | 463 |

**Supplementary Table 2.** Baseline characteristics of patients from each cohort individually.

|  | **PrediCOVID**  **(n = 133)** | **COVID19_OMICS-COVIRNA (n = 75)** | **TOCOVID**  **(n = 190)** | **MiRCOVID**  **(n = 65)** | ***P*** |
| --- | --- | --- | --- | --- | --- |
| Age (mean±SD) | 40.9±13.3 | 66.6±11.9 | 52.7±12.2 | 62.4±15.9 | 4.29E-40 |
| BMI (median[min,max]) | 25[19,39] | 27[20,52] | 28[18,57] | 30[14,53] | 2.92E-11 |
| Sex_male (n(%)) | 64(48.12) | 48(64.00) | 117(61.58) | 46(70.77) | 9.67E-03 |
| Smoker_curr (n(%)) | 23(17.29) | 5(6.67) | 9(4.74) | 10(15.38) | 7.39E-04 |
| Smoker_ex n(%)) | 25(18.80) | 7(9.33) | 23(12.11) | 4(6.15) | 0.0607 |

BMI: body mass index; SD: standard deviation. *P* value from ANOVA between the four cohorts is shown.
